# Diagnosis of Induced Resistance State in Tomato Using Artificial Neural Network Models Based on Supervised Self-Organizing Maps and Fluorescence Kinetics

**DOI:** 10.3390/s22165970

**Published:** 2022-08-10

**Authors:** Xanthoula Eirini Pantazi, Anastasia L. Lagopodi, Afroditi Alexandra Tamouridou, Nathalie Nephelie Kamou, Ioannis Giannakis, Georgios Lagiotis, Evangelia Stavridou, Panagiotis Madesis, Georgios Tziotzios, Konstantinos Dolaptsis, Dimitrios Moshou

**Affiliations:** 1 Laboratory of Agricultural Engineering, School of Agriculture, Aristotle University of Thessaloniki, 54124 Thessaloniki, Greece; 2 Laboratory of Plant Pathology, School of Agriculture, Aristotle University of Thessaloniki, 54124 Thessaloniki, Greece; 3 Institute of Applied Biosciences, Centre for Research and Technology Hellas, Thermi, 57001 Thessaloniki, Greece or; 4 Laboratory of Molecular Biology of Plants, School of Agricultural Sciences, University of Thessaly, 38221 Volos, Greece

**Keywords:** artificial intelligence, clustering, data mining, gene expression, plant protection

## Abstract

The aim of this study was to develop three supervised self-organizing map (SOM) models for the automatic recognition of a systemic resistance state in plants after application of a resistance inducer. The pathosystem *Fusarium oxysporum* f. sp. *radicis-lycopersici* (FORL) + tomato was used. The inorganic, defense inducer, Acibenzolar-S-methyl (benzo-[1,2,3]-thiadiazole-7-carbothioic acid-S-methyl ester, ASM), reported to induce expression of defense genes in tomato, was applied to activate the defense mechanisms in the plant. A handheld fluorometer, FluorPen FP 100-MAX-LM by SCI, was used to assess the fluorescence kinetics response of the induced resistance in tomato plants. To achieve recognition of resistance induction, three models of supervised SOMs, namely SKN, XY-F, and CPANN, were used to classify fluorescence kinetics data, in order to determine the induced resistance condition in tomato plants. To achieve this, a parameterization of fluorescence kinetics curves was developed corresponding to fluorometer variables of the Kautsky Curves. SKN was the best supervised SOM, achieving 97.22% to 100% accuracy. Gene expression data were used to confirm the accuracy of the supervised SOMs.

## 1. Introduction

Plant diseases are responsible for crop destruction and significant yield losses, which negatively affect the economy. It has been indicated that about 40% of crop losses occur due to plant diseases [1]. Taking into account that the population of the whole world is expected to increase up to 9 billion in the following years, production within the agricultural sector needs to be expanded by minimum of 70%, in order to meet food demands [2]. For this reason, early disease detection is regarded as an important tool for preventing low crop yields and consequently economic losses.

Traditional crop protection methods are usually based on crop inspections through visual observations, which is a limited, time consuming, and insufficient practice. The inconvenience of such practices lies in their weakness to discriminate the appearance of diseases with similar external symptoms. Recently, novel techniques for disease detection have been proposed that were proven to be capable of reducing misclassification and have been embedded in commercial platforms. Such techniques are employed by precision agriculture experts aiming to exploit attractive and efficient solutions, through computer vision or image processing tools of infected plants showing symptoms.

Among the large variety of crops, tomato is regarded as having significant economic importance, and this is the main reason that this plant is often used as a model plant for many research purposes [3,4].

Zhang et al. [5] proposed a three-channel convolution neural network (CNN) to detect tomato crop infections, including early blight, target spot, late blight, mosaic virus, bacterial speck, and Septoria leaf spot. The proposed CNN model reached an accuracy of 89.29%.

Kibriya et al. [6] demonstrated a deep learning approach, two (CNN) based models, namely GoogLeNet and VGG16, for tomato leaf disease recognition. The VGG16 GoogleNet and GoogLeNet models reached accuracies of 98%, and 99.23%, respectively, on the Plant Village dataset that comprised of a total of 10,735 leaf images.

However, solutions based on image processing have important limitations; for example, under field conditions, plant images are often noisy, which consequently leads to segmentation of a low quality, poor feature extraction, and then low model performance. Due to the high risk of incorrect analysis and diagnosis, alternative methods need to be explored that are both non-destructive and effective from every aspect of decision making.

Until the present, few studies have been carried out showing defense induction by microorganisms or chemical inducers. To overcome this, the present study introduces an original method to assess the success and outcome of defense-inducing applications in high value commercial crops such as tomato, which would favor the use of such strategies. The proposed method enables fast assessment of the effectiveness, decision-making, and adaptation of disease management schemes.

Induction of systemic resistance has been proposed as advantageous for reducing the severity of plant diseases [7]. The use of chemical plant defense inducers is an attractive, environmentally friendly means of plant protection [8,9]. However, several disadvantages, such as low reproducibility and effectiveness compared to chemical pesticides, and the difficulty to evaluate their efficacy in a timely manner, keep the use of resistance inducers in the background of the plant disease management artillery.

Biological defense activators, such as beneficial bacteria and other biological control agents [10], as well as several organic or inorganic defense inducers, have attracted interest. Acibenzolar-S-methyl (benzo-[1,2,3]-thiadiazole-7-carbothioic acid-S-methyl ester, ASM), an inorganic defense activator, is commercially available under the trade name Bion and has been proposed as a satisfactory means to induce resistance in tomato against various pathogens [11,12,13].

In plants, the induction of systemic resistance to pathogens may be triggered by the accumulation of various signaling molecules such as salicylic acid (SA) and production of pathogen-related proteins (PRs), or jasmonic acid (JA) and ethylene (ETH) [7]. To confirm the alerted defense state in plants, the expression of certain genes is studied, such as: *PAL*, which encodes phenylalanine ammonia-lyase, one of the key enzymes for producing salicylic acid [14]; *LOX* and *AOC*, which encode lipoxygenase and allene oxidase cyclase, respectively, two very important enzymes in the biosynthesis of jasmonic acid [15]; *PR1*, *PR3*, *PR5*, and *PR6*, which are response genes to salicylic acid accumulation and activation of the jasmonic acid/ethylene pathway [16,17].

Chlorophyll fluorescence imaging analysis has been used to analyze plant responses to abiotic and biotic stress factors, including pathogens and pests [18]. However, chlorophyll fluorescence imaging in primed plants has not yet been reported.

Unsupervised learning is a special form of learning that relies on clustering input data, without considering any output information. The data clustering attempts to approximate the probability density of the input data space by using a limited number of centroids. Self-organizing maps (SOMs) are widely applied artificial neural networks architectures [19] that rely on clustering and allocate the centroids according to a self-organizing learning algorithm, offering solutions that are often employed for various different unsupervised problems. An extension to self-organizing maps concerns supervised SOMs, which combine supervised and unsupervised learning. Their effectiveness is attributed to their special characteristic of combining both supervised and unsupervised learning techniques [20]. The counterpropagation artificial neural networks (CPANNs) share a common characteristic with SOMs, which is the additional layer that is appended to SOM [21]. Regarding classification problems, CPANNs have proven capable of performing nonlinear classification. Alterations to CPANNs layers have led to novel supervised models, including supervised Kohonen networks (SKNs) and XY-fused networks (XY-Fs) [22].

The combination of supervised SOM models and fluorescence kinetics data can be a revolutionary tool for diagnosing plant defense induction after the application of biological or other defense inducers. The induction of chlorophyll kinetic fluorescence has been shown to be a particularly sensitive indicator of various photosynthetic reactions, which are very important for understanding the various photosynthetic activities of plants [23]. These activities may indicate the existence of adverse effects of environmental changes, as well as resistance to various biotic and abiotic factors [24]. It is known that kinetic fluorescence curves (Kautsky curves) can be used to detect plant stress conditions or to classify plants in relation to their resistance to stress [25,26].

Automation and precision in agriculture has found many applications and can pave the way to future agricultural practices and production. However, automatic diagnosis of an induced resistance state in plants has not yet been reported. The current paper proposes an original method, through the development of three supervised SOM models for the automatic recognition of systemic resistance in plants, after application of resistance inducers. For the purpose of the study, the pathosystem *Fusarium oxysporum* f. sp. *radicis-lycopersici* (FORL) and tomato was used, and ASM was applied as an inducer. Three supervised SOM models were used to classify fluorescence kinetics data, in order to determine the induced resistance state in tomato plants. To achieve this, a parameterization of fluorescence kinetics curves was developed, corresponding to fluorometer variables of the Kautsky curves. Gene expression data were used to confirm the accuracy of the supervised SOMs. The proposed supervised SOM method employs three state-of-the-art algorithms for analyzing fluorescence kinetics, which have been used for the first time in this work to evaluate disease resistance in tomato plants. The current work is structured as follows: Section 1 (Introduction), where the physical problem, the state of the art, and the novelty of the current work are stated; Section 2 (Materials and Methods), where the experimental work and the three employed supervised SOMs methods are presented; Section 3 (Results and Discussion), where the efficiency of the proposed method is validated and the performances of the three applied supervised SOMs models are compared and contrasted; and Section 4 (Conclusions), where the results and the novelty of the proposed method are summarized based on the results presented in the Section 3 (Results and Discussion).

## 2. Materials and Methods

### 2.1. Production of Fusarium oxysporum f. sp. Radicis-Lycopersici and Inoculation

A virulent strain of FORL deposited in the Centraalbureau voor Schimmelcultures, The Netherlands (CBS 101587), was used for artificial inoculations of tomato plants. The protocol followed was described by Kamou et al. [27].

### 2.2. Growth and Inoculation of Tomato Plants

Plantlets of tomato cv. “Belladona”, at the 2-true leaf stage, grown in peat, in nursery trays, were transplanted into 100-mL pots, containing peat. Plants were grown for 7 days before challenge inoculation with FORL, to reach a leaf surface size that would allow fluorescence kinetics acquisition. The defense inducer ASM was applied 4 days after transplantation, and 72 h before challenge inoculation with FORL, by soil drenching. ASM solution was prepared in sterile distilled water, at a concentration of 25 mg/L (25 ppm), according to the dose recommended by the manufacturer, and 5 mL of solution was used per plant. Bion was applied in a single treatment. The pathogenic fungus was also applied as soil drenching, using 5 mL per plant of the spore suspension, prepared as described above. Plants that received no treatment were used as negative controls (hereafter referred to as control), whereas plants treated with FORL only, at 7 days after transplantation served as positive controls (hereafter referred to as FORL).

### 2.3. Fluorescence Kinetics Parameters

The chlorophyll fluorescence kinetics measurements were taken using a FluorPen FP 100-MAX-LM by SCI, which employs the OJIP protocol. Chlorophyll fluorescence kinetics were obtained at 48 and 72 h post challenge inoculation with FORL. For each of the 24 tomato plants, fluorescence parameters were acquired from the two middle leaves corresponding to treatment at 48 h, and the same procedure was followed for 12 tomato plants corresponding to the treatment at 72 h.

The chlorophyll fluorescence kinetics features extracted from the fluorescence parameters were used as training sets for the three different supervised SOMs introducing a novel, non-destructive method for recognizing the induced resistance condition in the Bion + FORL treatment. The proposed method was used to identify the other two investigated treatments, meaning the plants of the positive control (FORL) and the negative control (Control). Using the fluorometer, certain geometric parameters of the Kautsky curves were acquired, as described previously [28].

### 2.4. Supervised Self Organizing Maps (SOMs) Models

**Figure 1:**
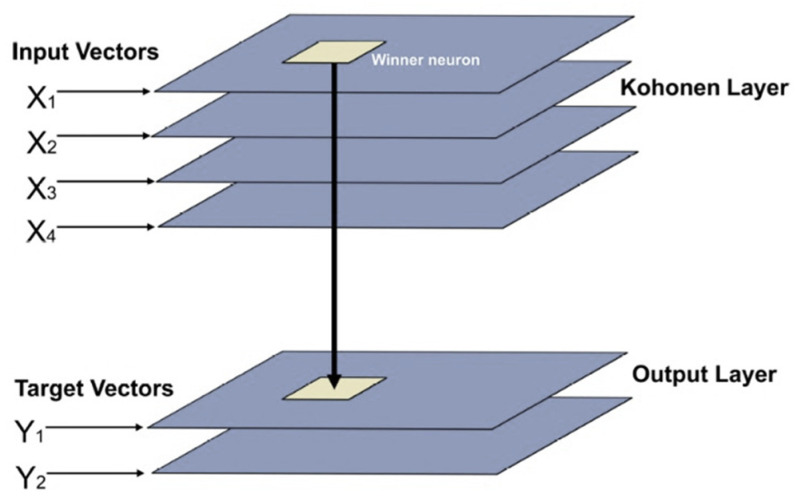
Structure of the CPANN model.

CPANN is considered a supervised SOM that augments the Kohonen layer, which performs the mapping of the input data, where the neurons are appended in a rectangular or hexagonal matrix, with the Grossberg layer, which acts as a pointing device. The constructed model consists of an input layer (Kohonen) and an output (Grossberg). The training procedure of CPANN is presented extensively in [20]. The CPANN is capable of generalizing on missing or faulty input data [22]. The Grossberg layer learns the target output values (T), while the Kohonen layer learns to estimate the mean input values. Figure 1 illustrates the structure of the CPANN model.

**Figure 2:**
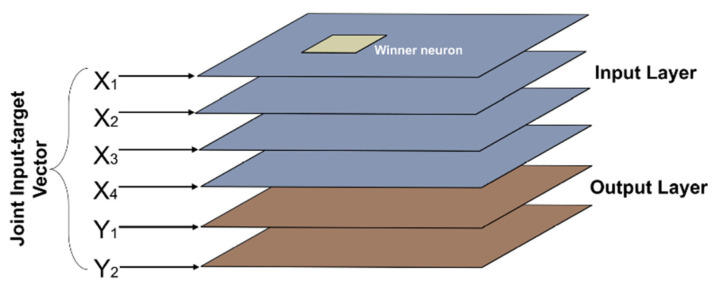
Structure of the SKN model.

In the SKN network, an input map (X_map_) and an output map (Y_map_) are combined, so as to produce a combined input and output map (XY_map_). Every input set X is linked to its corresponding output Y, so as to act in the form of an input data to XY_map_ [22]. The training procedure of SKN network is extensively presented in [20]. The SKN architecture differs from the two above mentioned supervised SOM models by augmenting the Kohonen and output layers into a joint entity, which is updated by using the training algorithm of SOMs. Figure 2 depicts the structure of the SKN model.

**Figure 3:**
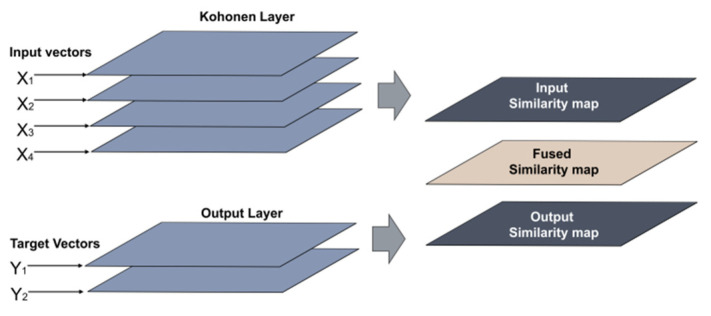
Structure of the XY-F model.

The XY-Fused network differs from SKN networks, mainly because it seeks similarities of both the input map (X_map_) and the output map (Y_map_) in a simpler manner. The training procedure of XY-F is extensively presented in [20]. The criterion for determining the winning neuron is a similarity identification, which is the result of weighing similarities with one object X and the other consisting units on the map X_map_, and similarities with the output objects Y and the units of the map Y_map_ [22]. Figure 3 illustrates the structure of the SKN model.

### 2.5. RNA Extraction from Tomato Plants and Relative Gene Expression Analysis

Samples of three leaves and the whole root were collected from three plants, randomly selected among the plants within each treatment, at 48 and 72 h after challenge inoculation with FORL (hpi), to be used for relative gene expression analysis.

Total RNA from the tomato leaves and roots was extracted from 3 biological replicates per treatment and time point, as described by Stavridou et al. [29]. Relative gene expression was assessed by quantitative reverse transcriptase PCR (RT-qPCR). The procedure, conditions, and primers used were as described by Stavridou et al. [29].

### 2.6. Statistical Analysis for Gene Expression

Gene expression analysis data were subjected to analysis of variance (ANOVA), based on completely randomized design (CRD), and mean values were computed from three replicates. Differences between treatment mean values were compared using Duncan’s test, and comparisons were made between treatments and the untreated control, at a significance level *p* ≤ 0.05. All statistical analyses were performed with SPSS v 25.0 software (SPSS Inc., Chicago, IL, USA).

## 3. Results and Discussion

### 3.1. Classification Results Obtained from the Fluorescence Data

The CPANN, SKN, and XY-F Networks were trained with the fluorescence-based parameters of the Kautsky Curves, in order to distinguish between the three treatments (Control-FORL-Bion + FORL). As mentioned in the Material and Methods (Section 2.3), a total of 24 plants were subjected to fluorescence acquisition for each treatment at 48 h and 12 plants for each treatment at 72 h, resulting in 36 plants per treatment that were combined into one dataset, comprising 36 plants per treatment. Gene expression at both time-points (Section 3.3) enabled this procedure. Cross validation was applied through dividing in a random manner the calibration data into four classes and keeping three classes for training, while validating on the fourth group. The aforementioned procedure was iterated for all possible combinations of the three classes. Then, the average result was acquired. Table 1, Table 2 and Table 3 show the performances of the three utilized supervised SOM models (SKN, XY-F, and CPANN) with a size equal to 12 × 12 neurons. The map sizes that were evaluated were rectangular, with 9, 25, 64, 100, 144, and 225 neurons. A common observation was that with increasing size, the results tended to improve, up to 144 neurons, and after that the results decreased. The best results were obtained for a (144 neurons) grid.

The comparative results of the three supervised SOM models, which are presented in the above Table 1, Table 2 and Table 3, indicate that the best performing was the SKN model, which reached an accuracy of 97.22% for treatments 2 and 3, while for treatment 1, it reached an accuracy of 100%.

### 3.2. Confirmation of the Supervised SOM Accuracy in Prediction of the Prime State of Plants with Gene Expression Data

**Figure 4:**
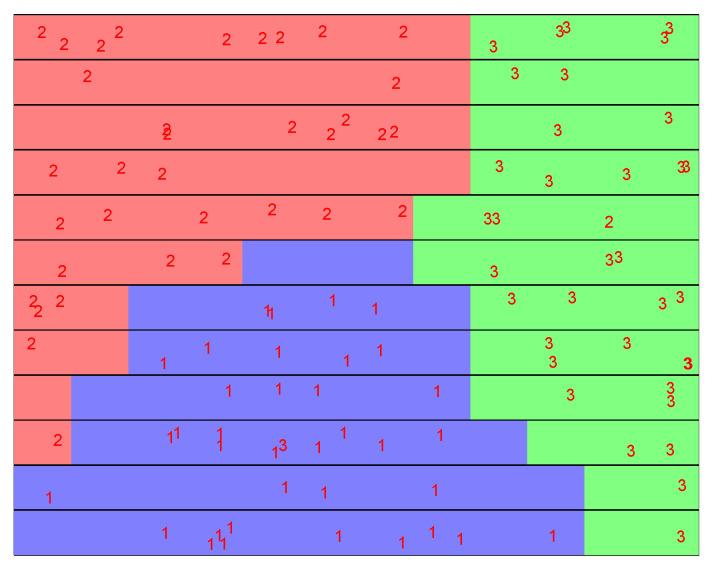
The supervised SOM clusters demonstrates the discrimination between the three applied treatments, represented as class 1 (Control, colored in purple), class 2 (FORL colored in red), and class 3 (Bion + FORL, colored in green).

Figure 4 shows clusters formed on a 12 × 12 SKN supervised SOM, where the samples are shown and where the results of Table 3 clearly show that one sample from class 3, Bion + FORL, was misclassified as class 1, Control, while a sample from class 2, FORL, was misclassified as Bion + FORL treated.

### 3.3. Gene Expression Analysis in Tomato Plants Challenged with FORL, 48 and 72 h after Induction Treatment with Bion

**Figure 5:**
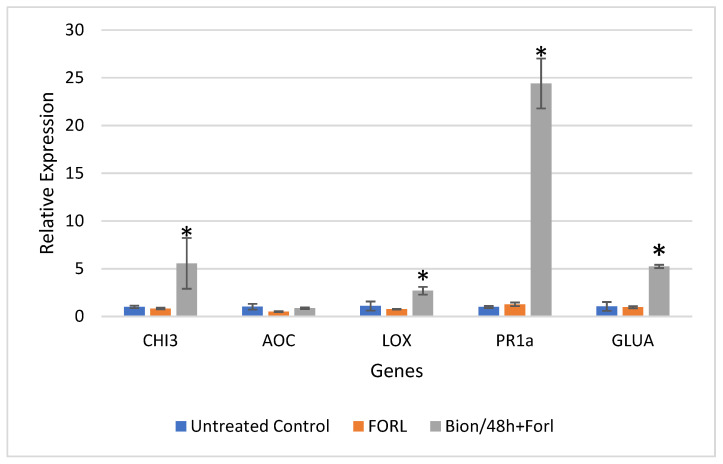
Analysis of defense genes *CHI3*, *AOC*, *LOX*, *PR-1a,* and *GLUA* in tomato leaves challenged with *Fusarium oxysporum* f. sp. *radicis-lycopersici* (FORL), after induction treatment with Bion, at 48 hpi. Error bars indicate the variation based on three biological replicates. The asterisk denotes substantial deviation with respect to the control treatment, according to Duncan’s test, *p* ≤ 0.05.

**Figure 6:**
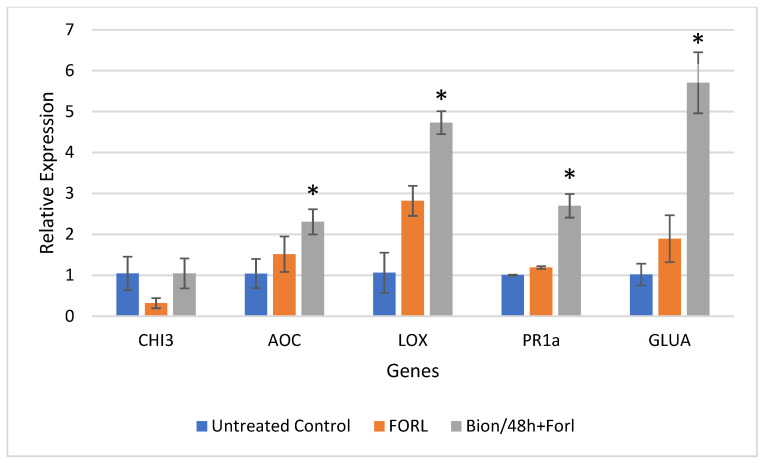
Analysis of defense genes *CHI3*, *AOC*, *LOX*, *PR-1*a, and *GLUA* in tomato roots challenged with *Fusarium oxysporum* f. sp. *radicis-lycopersici* (FORL), after induction treatment with Bion, at 48 hpi. Error bars indicate the variation based on three biological replicates. The asterisk denotes substantial deviation with respect to the control treatment, according to Duncan’s test, *p* ≤ 0.05.

**Figure 7:**
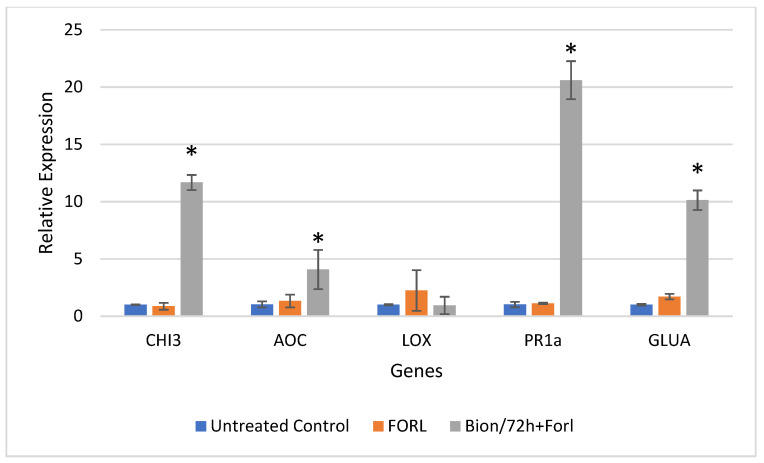
Analysis of defense genes *CHI3*, *AOC*, *LOX*, *PR-1*a, and *GLUA* in tomato leaves challenged with *Fusarium oxysporum* f. sp. *radicis-lycopersici* (FORL), after induction treatment with Bion, at 72 hpi. Error bars indicate the variation based on three biological replicates. The asterisk denotes substantial deviation with respect to the control treatment, according to Duncan’s test, *p* ≤ 0.05.

**Figure 8:**
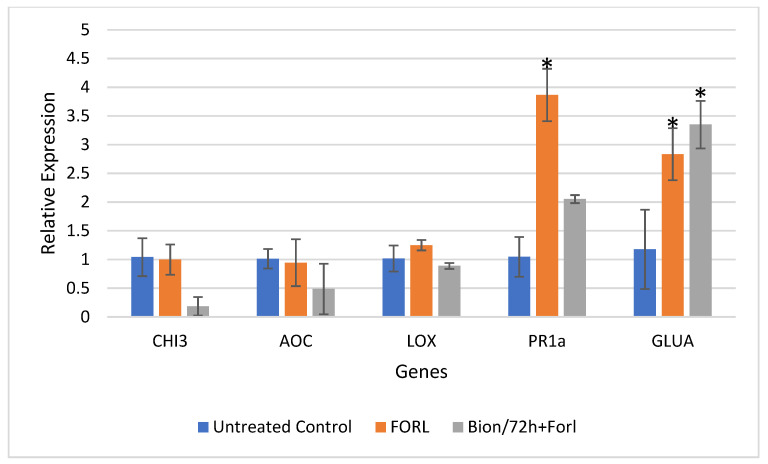
Analysis of defense genes *CHI3*, *AOC*, *LOX*, *PR-1a,* and *GLUA* in tomato roots challenged with Fusarium oxysporum f. sp. radicis-lycopersici (FORL), after induction treatment with Bion, at 72 hpi. Error bars indicate the standard deviation variation based on three biological replicates. The asterisk indicates denotes substantial deviation with respect to the control treatment, according to Duncan’s test, *p* ≤ 0.05.

Results of the relative gene expression in the leaves and roots of tomato plants challenged with FORL, 48 and 72 hpi, are presented for all treatments in Figure 5, Figure 6, Figure 7 and Figure 8.

The data indicated a successful priming of the plants. Figure 5 and Figure 6 present the relative gene expression analysis in leaves and roots of tomato, respectively, at 48 hpi. Comparisons are made with the Control, and statistical differences are indicated with an asterisk. More specifically, regarding the expression in tomato leaves, at 48 hpi, a 5.54-fold, 24.36-fold, and 4.96-fold induction of *CHI3*, *PR-1a*, and *GLUA*, respectively, was observed, after challenge inoculation with FORL, when Bion was applied, as compared to the untreated control (Figure 5). Regarding the same treatment and the expression in tomato roots, the genes *LOX*, *PR-1a*, and *GLUA* exhibited an induction in expression of 4.46-fold, 2.68-fold, and 5.6-fold, respectively (Figure 6).

Figure 7 and Figure 8 present the relative gene expression analysis in leaves and roots of tomato, respectively, at 72 hpi. Comparisons are made with the Control, and statistical differences are indicated with an asterisk. Regarding the expression in tomato leaves, at 72 hpi, the mean transcript levels of genes *CHI3*, *AOC*, *PR1-a*, and *GLUA* were higher by 11.6-fold, 3.95-fold, 20.16-fold, and 10.14-fold, respectively, in the Bion + FORL treatment, as compared to the untreated control (Figure 4). Whereas, in the tomato roots, *PR1-a* and *GLUA* were both induced by 3.7-fold and 2.41-fold, respectively, by exposure to the pathogen alone (Figure 8). These two genes were also slightly upregulated (1.96-fold and 2.85-fold, respectively), after treatment with Bion and challenge inoculation with FORL (Figure 8).

Plant defense response to pathogens is characterized by various physiological, biochemical, and molecular changes [30]. Diagnosis of such changes is laborious, time-consuming, expensive, and requires plant tissue destruction. A non-destructive, instant method that allows fast and accurate diagnosis of the induction state would open a new perspective in plant priming for disease treatment. Alternatives to fungicidal control means against plant diseases are expected to have an important implication for plant pathogen management. Chemical defense inducers act indirectly against the pathogens, by promoting the orchestration of defense reactions in the plants that are governed by expression of defense related genes [7]. Fast monitoring of this expression in vivo represents a pioneering tool for exploitation of such disease management practices, since it would promote their use instead of chemicals and would allow early assessment of their effectiveness and timely decision-making.

In the present study, several genes reported to be related to defense responses in tomato [27,31,32,33] were used to study the defense induction state achieved after treatments. Variable expression of *CHI3*, *AOC*, *LOX*, *PR-1a*, and *GLUA* was confirmed after application of ASM and FORL in tomato leaves and roots, demonstrating the induction of resistance in the plants with these treatments. The presented approach confirmed the original hypothesis, according to which, physicochemical and molecular alterations in the alerted plant organism have an impact on fluorescence kinetics, as shown in other cases of biotic and abiotic stress, including pathogens and pests [18]. The novel combination of supervised SOM models with fluorescence kinetics was proven to be able to recognize different harvesting stages in lettuce plants [28]. The original contribution of the current work is the non-destructive diagnosis of the induced resistance state of a plant. Different expression profiles among treatments, especially, FORL, and Bion + FORL confirmed the differences in fluorescence kinetics. 

The combination of in vivo fluorescence techniques with supervised SOM models can form a cost-efficient, yet effective, solution in the field of plant protection, since it is non-invasive and applicable to all plants, at any time. The ability of supervised SOMs to exploit the class separation that is already exhibited by the fluorescence feature is achieved by tuning their weights to reflect this discriminative behavior. Moreover, their supervised character allows the quantification of the class assignment into a crisp classification result.

More precisely, in the case of the SKN network, which demonstrated the best performance of all the supervised SOM models, the input and output layers are gathered together, so as to form a combined layer. This layer tends to scale according to the nature of the training scheme. Thus, this behavior gives to the SKN network a more tunable character compared to the two other networks employed (XY-F, CPANN), which explains its successful classification rates.

The combined approach of supervised SOM models and fluorescence kinetics data for the diagnosis of induced resistance state is capable of providing a useful tool in the field of plant protection and is subsequently expected to have a long-term impact on sustainable agricultural production, by improving the effectiveness of environmentally friendly plant disease control measures, through an early assessment of their effectiveness, facilitating decision-making and reducing the use of agrochemicals.

## 4. Conclusions

In the current study, three supervised SOM models, namely SKN, CPANN, and XY-F were employed for the automatic recognition of systemic resistance in tomato plants after application of a resistance inducer. Fluorescence parameterization was developed, corresponding to the fluorometer variables of Kautsky curves. Gene expression data were used to verify the accuracy of the supervised SOMs. The successful recognition of three different defense induction treatments with the help of the supervised SOMs was achieved through taking advantage of the heterogeneous nature of the fluorescence kinetics parameters. The performance of the supervised SOM models was high, reaching accuracies from to 88.89% to 100%, demonstrating the effectiveness of the proposed automatic recognition method. It was indicated that the supervised SOMs ability for diagnosing plant defense induction is attributable mostly to the fluorescence features’ tendency to form clusters that are closely related to the occurrence of defense induction. Operational application of the supervised SOMs models is dependent on the deployment of the trained models under controlled conditions, so as to monitor the expression of resistance in tomato plants. The indication of specific fluorescence features with close correlations to defense induction offers an opportunity for precise classification, with less fluorescence features included, introducing an effective, yet non-destructive, solution for plant pathogen management.

## Figures and Tables

**Table 1:** Accurate identification for each of the three applied treatments from the SKN network architecture based on the fluorescence kinetics features [28].

Real Treatment Class	Accurate Identification of Treatment 1 (Control)	Accurate Identification of Treatment 2 (FORL)	Accurate Identification of Treatment 3 (Bion + FORL)
**Control**	100%	0%	0%
**FORL**	0%	97.22%	2.78%
**Bion + FORL**	2.78%	0%	97.22%

**Table 2:** Accurate identification of each of the three applied treatments from the CPANN network architecture, based on the fluorescence kinetics features [28].

Real Treatment Class	Accurate Identification of Treatment 1 (Control)	Accurate Identification of Treatment 2 (FORL)	Accurate Identification of Treatment 3 (Bion + FORL)
**Control**	97.22%	2.78%	0%
**FORL**	11.11%	88.89%	0%
**Bion + FORL**	5.56%	8.33%	86.11%

**Table 3:** Accurate identification of each the three applied treatments from the XY-F network architecture, based on the fluorescence kinetics features [28].

Real Treatment Class	Accurate Identification of Treatment 1 (Control)	Accurate Identification of Treatment 2 (FORL)	Accurate Identification of Treatment 3 (Bion + FORL)
**Control**	100%	0%	0%
**FORL**	8.33%	88.89%	2.78%
**Bion + FORL**	0%	2.78%	97.22%
